# Atomic Diffusivities of Yttrium, Titanium and Oxygen Calculated by Ab Initio Molecular Dynamics in Molten 316L Oxide-Dispersion-Strengthened Steel Fabricated via Additive Manufacturing

**DOI:** 10.3390/ma17071543

**Published:** 2024-03-28

**Authors:** Zhengming Wang, Seongun Yang, Stephanie B. Lawson, V. Vinay K. Doddapaneni, Marc Albert, Benjamin Sutton, Chih-Hung Chang, Somayeh Pasebani, Donghua Xu

**Affiliations:** 1 Materials Science Program, Oregon State University, Corvallis, OR 97331, USA; wangz6@oregonstate.edu (Z.W.); chih-hung.chang@oregonstate.edu (C.-H.C.); 2 School of Mechanical, Industrial and Manufacturing Engineering, Oregon State University, Corvallis, OR 97331, USA; yangseo@oregonstate.edu (S.Y.); lawsonst@oregonstate.edu (S.B.L.); somayeh.pasebani@oregonstate.edu (S.P.); 3 Advanced Technology and Manufacturing Institute (ATAMI), Corvallis, OR 97330, USA; doddapav@oregonstate.edu; 4 School of Chemical, Biological and Environmental Engineering, Oregon State University, Corvallis, OR 97331, USA; 5 Electric Power Research Institute (EPRI), Charlotte, NC 28262, USA; malbert@epri.com (M.A.); bsutton@epri.com (B.S.)

**Keywords:** diffusivity, additive manufacturing, ab initio molecular dynamics, oxide-dispersion-strengthened alloys, machine learning force field

## Abstract

Oxide-dispersion-strengthened (ODS) steels have long been viewed as a prime solution for harsh environments. However, conventional manufacturing of ODS steels limits the final product geometry, is difficult to scale up to large components, and is expensive due to multiple highly involved, solid-state processing steps required. Additive manufacturing (AM) can directly incorporate dispersion elements (e.g., Y, Ti and O) during component fabrication, thus bypassing the need for an ODS steel supply chain, the scale-up challenges of powder processing routes, the buoyancy challenges associated with casting ODS steels, and the joining issues for net-shape component fabrication. In the AM process, the diffusion of the dispersion elements in the molten steel plays a key role in the precipitation of the oxide particles, thereby influencing the microstructure, thermal stability and high-temperature mechanical properties of the resulting ODS steels. In this work, the atomic diffusivities of Y, Ti, and O in molten 316L stainless steel (SS) as functions of temperature are determined by ab initio molecular dynamics simulations. The latest Vienna Ab initio Simulation Package (VASP) package that incorporates an on-the-fly machine learning force field for accelerated computation is used. At a constant temperature, the time-dependent coordinates of the target atoms in the molten 316L SS were analyzed in the form of mean square displacement in order to obtain diffusivity. The values of the diffusivity at multiple temperatures are then fitted to the Arrhenius form to determine the activation energy and the pre-exponential factor. Given the challenges in experimental measurement of atomic diffusivity at such high temperatures and correspondingly the lack of experimental data, this study provides important physical parameters for future modeling of the oxide precipitation kinetics during AM process.

## 1. Introduction

As the expectations grow for the properties of next-generation alloys that can be used in harsh environments, oxide-dispersion-strengthened (ODS) alloys are gradually emerging as a promising candidate due to their excellent creep resistance, corrosion resistance, and high-temperature tensile properties and thermal stability [1,2,3,4,5,6,7,8,9,10,11]. ODS alloys are reinforced by numerous nano-sized oxide particles, such as Y_2_O_3_, Y_2_TiO_5_ or Y_2_Ti_2_O_7_, that are uniformly dispersed in the matrix and responsible to impede dislocation motion.

Conventionally, ODS alloys are fabricated through time-consuming mechanical alloying (high energy ball milling) and subsequent heat treatments such as hot isostatic pressing (HIP) and hot rolling [11,12,13]. Micro-sized oxide particles are first broken down into smaller sizes and the oxide elements are dissolved into the alloy matrix during the mechanical alloying. During the heat treatments, the elements precipitate out from the alloy matrix as nano-sized oxide particles. All these processes occur in the solid state, which has rather limited atomic mobility (diffusivity). More recently, laser-melting-based additive manufacturing (AM) technologies, such as laser powder bed fusion (LPBF) and laser direct energy deposition (LDED), have been demonstrated to be able to directly print ODS alloys with lightly mixed matrix powder (or wire) and oxide particles [14,15,16,17]. The oxide dispersion elements from the feedstock are dissolved in the melt pool and then precipitate out as oxide nano particles during rapid solidification. Dissolution and precipitation occur almost instantaneously, which brings great promise for reducing the labor, energy and cost associated with manufacturing ODS alloys.

The mechanical properties of ODS alloys are primarily controlled by the number density, composition and size of the oxide precipitates, which, in AM, vary with the process parameters, such as laser power and scanning speed [14,15,16,17,18,19,20,21]. Hence, it is important to establish a clear correlation between the oxide characteristics and the AM process parameters. Multiscale modeling of the precipitation kinetics based on the fundamental physics (diffusion, clustering and competing re-dissolution) can reduce the amount of experimental effort towards this end. Performing such modeling requires atomic diffusivities of the oxide dispersion elements (here Y, Ti and O) in the molten metallic matrix (here 316L SS) over a wide temperature window. However, measuring atomic diffusivities at the relevant high temperatures is extremely challenging, leading to the scarcity of such experimental data. Meanwhile, it is also challenging to compute atomic diffusivity in molten multi-element alloy due to the complex composition, the disordered liquid structure and significant thermal vibration. To the best of our knowledge, there have been no reports of experimentally or computationally determined diffusivities of Y, Ti or O in molten 316L SS, although some studies have been performed to determine diffusivities of Y, Ti or O in body-centered cubic (BCC) iron or molten iron through experiments or computation [22,23,24,25,26,27,28,29,30,31,32]. For example, the O-diffusivity in molten pure iron and Ti-diffusivity in molten carbon-saturated iron have been measured by different experimental methods slightly above the melting temperature (*T*_m_) [22,23,24,25]. Some density functional theory (DFT) and kinetic Monte Carlo (kMC) simulations have been conducted to obtain Y-, Ti- or O-diffusivity in BCC iron [26,27,28,29,30,31,32].

In this study, we calculate the diffusivities of Y, Ti, and O in the molten 316L SS using ab initio molecular dynamics (AIMD). AIMD combines the Schrodinger-equation-based electronic structure calculation of the ab initio method with the Newtonian law-based atomic trajectory prediction of molecular dynamics (MD). Unlike typically performed 0K pure ab initio calculations, AIMD allows for thermal vibration, spontaneous structural evolution and sampling over a wide range of disordered atomic configurations in the liquid (molten) state. To enhance the efficiency of AIMD, we use the new feature of the on-the-fly machine learning force field in the latest VASP package. More details about our methodology are provided in the next section, following which we will present the directly computed diffusivity values for individual temperatures and the fitted Arrhenius expressions containing the activation energy and pre-exponential factor for Y, Ti and O diffusion in the molten 316L SS. The Arrhenius expressions can be coded directly into the future kinetic models to supply the diffusivities of Y, Ti and O in the melt pool of the 316L SS at any temperature (above T_m_) during its cooling process. 

## 2. Methodology

### 2.1. VASP Setup

The VASP software (version 6.3.2) [33,34,35,36] is employed in this work to perform AIMD simulations of the diffusion of Y, Ti, and O in molten 316L SS on the high-performance computing (HPC) cluster of College of Engineering, Oregon State University. For all the simulations, we use the NPT (controlled number of atoms N, pressure P, and temperature T) ensemble, the pseudopotentials generated with the projector-augmented wave (PAW) method and the generalized gradient approximation (GGA) based on the Perdew–Burke–Ernzerhof (PBE) functional [37,38], a plane-wave cutoff energy of 400 eV, and a uniform Monkhorst–Pack k-mesh (2 × 2 × 2, automatic generation). The temperature and pressure are controlled by the Langevin thermostat and the Parinello–Rahman barostat, respectively. Post-simulation 3D visualization and data analysis are performed with Open Visualization Tool (OVITO) (version 3.8.4) [39] and Matlab (version R2023b).

#### 2.1.1. The Machine Learning Force Field (MLFF)

Traditionally, AIMD simulations are very computationally expensive as they involve repeated electronic calculations every MD step of the simulated dynamic process. Starting from Ver. 6.3.0, VASP has implemented a new feature—an on-the-fly machine learning force field. This new feature allows VASP to build an MLFF (that quantitatively describes the atomic-level interactions among the relevant elements) by learning from some initial integrated ab initio and MD steps, and then the user can apply the MLFF to perform only MD in later simulations, skipping the time-consuming ab initio steps [33,34,35,36]. The machine learning steps do not have to use exactly the same dynamic process, material composition, or system size as in the later application simulations. 

**Figure 1:**
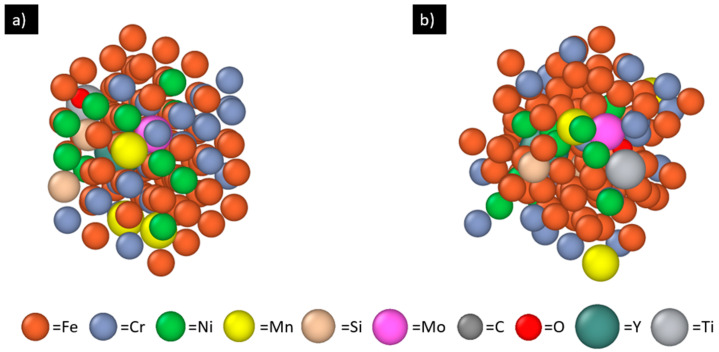
The AIMD model with 128 atoms for training/building the MLFF: (**a**) initial configuration of 316L SS with one Y atom, one Ti atom, and one O atom; (**b**) molten 316L SS with one Y atom, one Ti atom, and one O atom after learning process.

In this work, we perform machine learning of the force field with a supercell comprising 128 atoms (note that it is quite typical for ab initio calculations to use fewer than one hundred atoms in order to reduce the computational cost). To approximate the nominal 316L SS composition (listed in Table 1) a 4 × 4 × 4 BCC Fe supercell (128 atoms) is first created and 42 of the Fe atoms are then randomly replaced with the other constituent elements (e.g., Cr, Ni, Mn) according to their target atomic percentages (exact numbers of atoms for the elements are listed in Table 1). Due to the very low atomic percentages of phosphorus and sulfur, these two elements are not included in this work. Then, one Y, one Ti, and one O atom are incorporated into the system by replacing three of the remaining Fe atoms randomly. This system (shown in Figure 1a) is then heated from 1700 K to 2700 K in 10 picoseconds (with a step size of 2 femtoseconds) to train/build the MLFF. During the heating, the crystal lattice is melted into a disordered liquid structure (shown in Figure 1b) as expected. Thus, the resulting MLFF is appropriate for treating atomic interactions among all the involved elements in the molten 316L SS matrix. The Bayesian error estimation for energy per atom during the 5000 learning steps (with temperature increasing from 1700 K to 2700 K) is provided in the Supplementary Materials. The error grows slowly with increasing steps (i.e., temperature) but overall well contained within a few times 10^−5^ eV/atom.

#### 2.1.2. Diffusion Simulation

**Figure 2:**
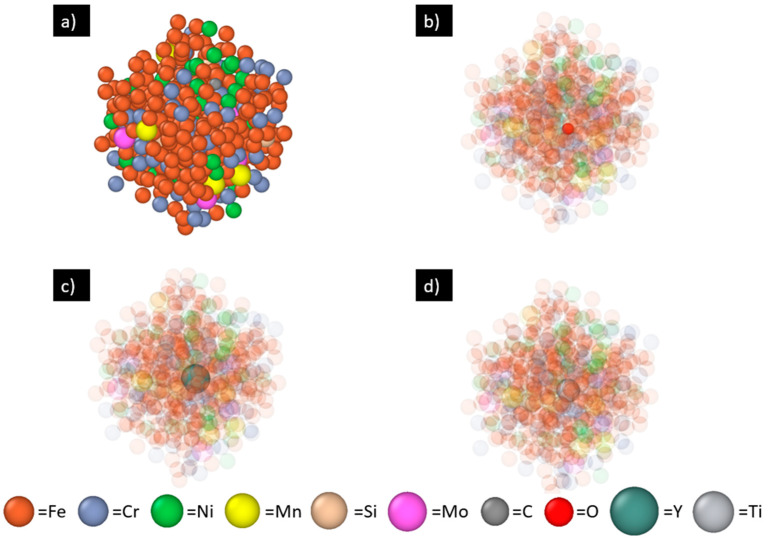
One representative AIMD model with 432 atoms for diffusion simulation: (**a**) the matrix—the molten 316L SS sample S1, (**b**) S1 with one O atom (solid red), (**c**) S1 with one Y atom (solid dark green), and (**d**) S1 with one Ti atom (solid grey). Matrix atoms are made translucent in (**b**–**d**).

After the MLFF is built, diffusion simulation (with MD steps only) is performed using a larger system size containing 432 atoms. A 6 × 6 × 6 BCC Fe supercell is first created and annealed at 2200 K until completely melted. Then, 140 Fe atoms are randomly replaced by Cr, Ni, Mn, etc., according to the 316L SS composition (exact numbers of atoms for the elements are listed in Table 1—“No. of atoms for diffusion simulations”). For better statistics, this random replacement is performed three times here, creating three molten 316L SS samples S1–S3 with different initial atomic configurations. Next, for each 316L SS sample, one Y, Ti, or O atom is then introduced to the system by replacing one extra Fe atom randomly, as shown in Figure 2. Then, the 9 diffusion systems (3 matrices: S1, S2 and S3; 3 diffusing species: Y, Ti, O) are each annealed at four different temperatures, 1850 K, 2000 K, 2200 K, and 2500 K, for 20 picoseconds with a step size of 2 femtoseconds. The time-dependent coordinates of all the atoms (including Y, Ti or O atom and the matrix atoms) are saved every 40 femtoseconds in the XDATCAR file. The first 8 picoseconds of each simulation are treated as system relaxation, and the data in that timeframe are not used for diffusivity calculation.

### 2.2. Diffusivity Calculation

After each simulation, the XDATCAR file containing the coordinates of all the atoms is read into OVITO and then the coordinates of the target Y, Ti or O atom are selected out and exported into a separate file together with the time steps. The mean square displacement (MSD) of the diffusing atom is then calculated with Matlab:

(1) $$\mathrm{MSD}\left(i\right)=\frac{1}{n-i}\sum_{j=1}^{n-i}\left\{{\left[x\left(j+i\right)-x\left(j\right)\right]}^{2}+{\left[y\left(j+i\right)-y\left(j\right)\right]}^{2}+{\left[z\left(j+i\right)-z\left(j\right)\right]}^{2}\right\}$$

where n is the total number of time steps, i is the i-th time step (representing the diffusion time), x(j+i), y(j+i) and z(j+i) are the coordinates of the atom at the (j+i)-th time step, and x(j), y(j) and z(j) are the coordinates at the j-th time step. Then, the diffusivity in the 3-D space is calculated by the Einstein–Smoluchowski equation:

(2) $$D=\frac{\mathrm{MSD}\left(t\right)}{6t}=\frac{\mathrm{MSD}\left(i\right)}{6\times i\times stepsize}$$

For each diffusing species (Y, Ti or O), the diffusivity values obtained from S1, S2 and S3 samples at a common temperature are averaged and then the averaged values at different temperatures are fitted to the Arrhenius equation:

(3) $$D\left(T\right)=D_{0}\exp\left(-\frac{E_{a}}{RT}\right)$$

where $D_{0}$ is the pre-exponential factor, $E_{a}$ is the activation energy, R is the universal gas constant and T is the absolute temperature. 

## 3. Results and Discussion

### 3.1. Diffusivity of Oxygen

**Figure 3:**
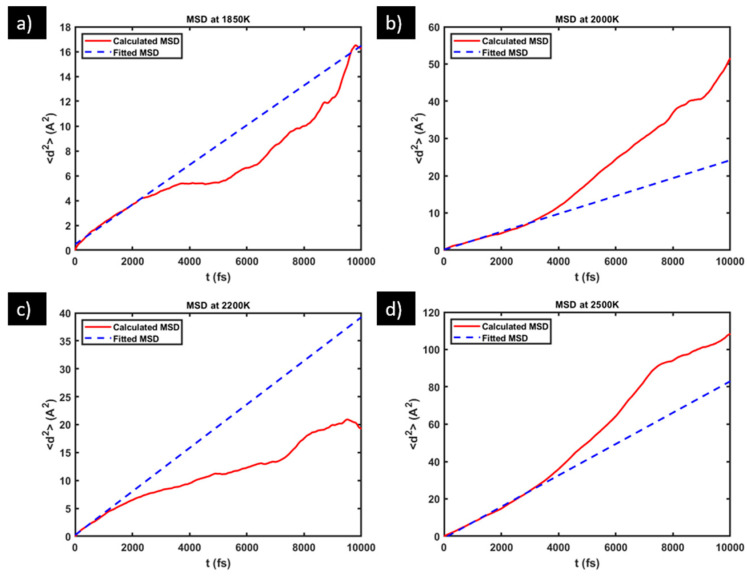
Plot (red, solid) of MSD vs. diffusion time in sample S1 and the fitting (blue, dashed) of its initial linear segment at: (**a**) 1850 K, (**b**) 2000 K, (**c**) 2200 K, and (**d**) 2500 K.

The red solid curves in Figure 3 are the plots of the MSD of O in the molten 316L SS (sample S1) vs. diffusion time at the four different temperatures. All the MSD plots exhibit an initial linear segment, followed by erratic deviations from the linear behavior. This characteristic is well known for MSD plots based on any finite number of time steps (and corresponding coordinates), whether in simulations or experiments. The initial linear segment follows the theoretical expectation as depicted by Equation (2) very closely, owing to the sufficient statistics in the averaging over *j* for a fixed *i* in Equation (1), when *i* is small. The later erratic deviation from the linear behavior is caused by two factors: less data points going into the averaging in Equation (1), and more correlation among the displacement steps that are being averaged. However, this is not a problem for obtaining the correct diffusivity; it just means that one should focus on the initial linear segment of an MSD curve where the MSD data carry the highest statistical significance, provided that it has been long enough to capture the diffusive stage of atomic displacements. 

**Figure 4:**
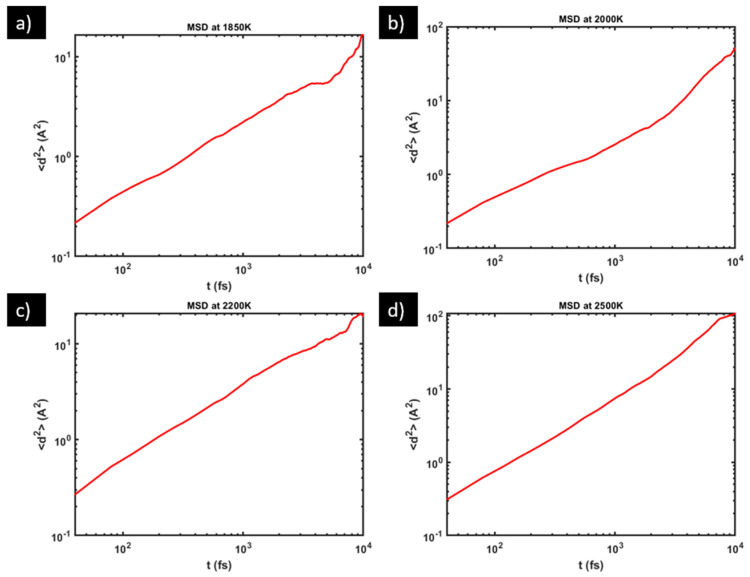
Log–log plot of MSD vs. diffusion time in sample S1 at: (**a**) 1850 K, (**b**) 2000 K, (**c**) 2200 K, and (**d**) 2500 K.

One way to judge if the MSD data have captured the diffusive stage is to use the log–log plot of MSD vs. time. On a full-spectrum log–log plot, MSD generally shows three stages: the ballistic stage (*t*^2^ dependence, slope ≈ 2) at the shortest time scale, followed by a plateau stage (*t*^0^ dependence, slope ≈ 0) at the intermediate time scale, and then by the diffusive stage (*t*^1^ dependence, slope ≈ 1) at the longer time scale. The exact time scales for the different stages vary with the material system and with temperature. Here, in Figure 4, we show the log–log version of the MSD vs. time plots for O diffusion in sample S1 (whose linear version is presented in Figure 3). These log–log plots show a fairly stable slope ≈ 1 over more than one order of magnitude (40 fs to ~1500 fs, where 40 fs is the time interval between two outputs of coordinates in our VASP setting, that is, the smallest time interval recorded for MSD calculations). This evidences that our MSD data have indeed captured the diffusive stage of atomic displacements, even for the smallest recorded time interval 40 fs. The absence of the ballistic stage and the plateau stage in Figure 4 is attributable to the high temperatures in this study at which the time scales of those stages fall below the minimum recorded time interval 40 fs. 

Having ensured that the early segment of the MSD vs. time data have already captured the diffusive stage, we fit the initial segments (40 to ~1500 fs) of all the MSD plots in Figure 3 into a linear function, as shown by the blue dashed lines therein, and use the fitted slope in place of the $\frac{\mathrm{MSD}\left(t\right)}{t}$ term in Equation (2) to calculate the diffusivity. Table 2 lists the O-diffusivity values at the four different temperatures obtained from the three different samples S1–S3. 

**Figure 5:**
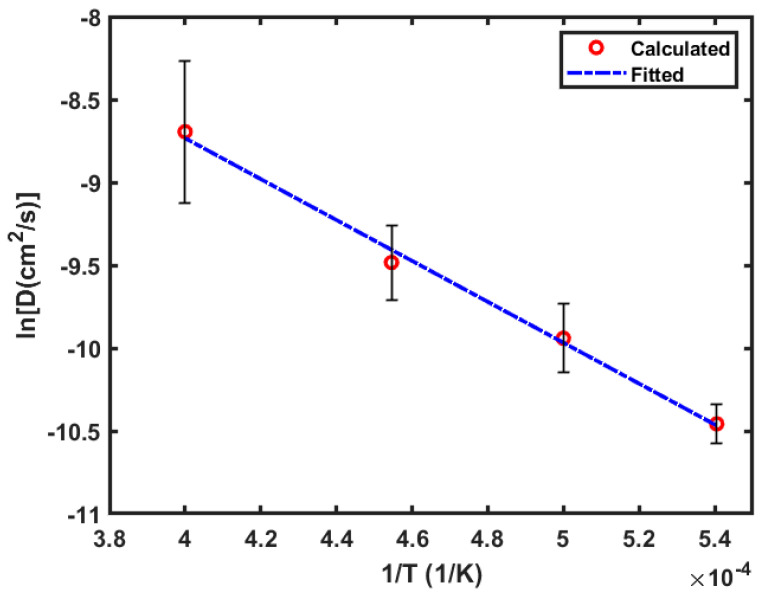
Calculated average diffusivity (red circles), and its Arrhenius fitting (blue dashed line) for oxygen in molten 316L SS. Note that the error bars represent the differences among the calculated diffusivities from the three samples S1–S3 at each temperature before averaging, and they are not related to the fitting of the averaged diffusivity.

The averaged O-diffusivity at 1850 K, 2000 K, 2200 K, and 2500 K is 2.89 × 10^−5^ cm^2^/s, 4.88 × 10^−5^ cm^2^/s, 7.74 × 10^−5^ cm^2^/s, and 1.79 × 10^−4^ cm^2^/s, respectively. They are plotted (red circles) in Figure 5 in terms of ln(D) vs. $\frac{1}{T}$. According to the Arrhenius equation (Equation (3)), the two quantities should have a linear correlation, $\ln\left(D\right)=\ln\left(D_{0}\right)-\frac{E_{a}}{R}\frac{1}{T}$. By fitting the ln(D) vs. $\frac{1}{T}$ data, one can determine the $D_{0}$ and Q. As displayed by the blue dashed line in Figure 5, the fitting captures the trend in the data very well. This is also evidenced by a high fitting goodness $R^{2}=0.993$. From the fitting, the pre-exponential factor is determined to be $D_{0}=2.78\times 10^{-2}$ cm^2^/s, and the activation energy to be $E_{a}=1.06\times 10^{5}$ J/mol. Hence, the O-diffusivity ($D_{O}$) in the molten 316L SS has the Arrhenius form of

(4) $$D_{O}\left(T\right)=2.78\times 10^{-2}\exp\left(-\frac{1.06\times 10^{5}}{RT}\right)$$

### 3.2. Diffusivities of Yttrium and Titanium

**Figure 6:**
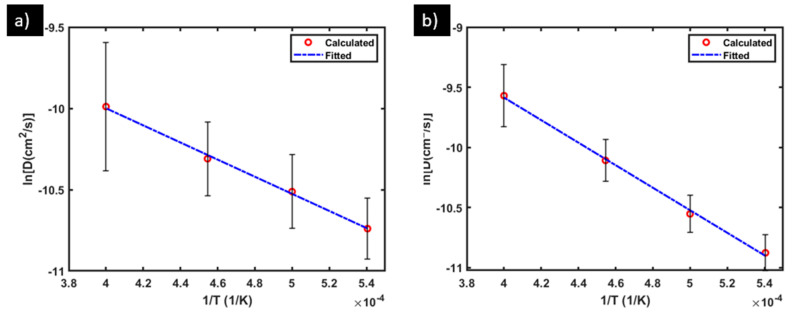
Calculated average diffusivity (red circles), and its Arrhenius fitting (blue dashed line) for (**a**) Y and (**b**) Ti in molten 316L SS. Note that the error bars represent the differences among the calculated diffusivities from the three samples S1–S3 at each temperature before averaging, and they are not related to the fitting of the averaged diffusivity.

Following the same procedure described in Section 3.1, the VASP simulation data for Y and Ti diffusion in the molten 316L SS are analyzed. As listed in Table 3, the Y-diffusivity averaged over the three different samples is: 2.19 × 10^−5^ cm^2^/s, 2.77 × 10^−5^ cm^2^/s, 3.39 × 10^−5^ cm^2^/s, and 4.85 × 10^−5^ cm^2^/s at 1850 K, 2000 K, 2200 K, and 2500 K, respectively. They are plotted (red circles) in Figure 6a and fitted (blue dashed line, Figure 6a) into the Arrhenius form of

(5) $$D_{Y}\left(T\right)=4.40\times 10^{-4}\exp\left(-\frac{4.62\times 10^{4}}{RT}\right)$$

with a high fitting goodness $R^{2}=0.994$. 

The averaged Ti-diffusivity, also listed in Table 3, is: 1.90 × 10^−5^ cm^2^/s, 2.63 × 10^−5^ cm^2^/s, 4.11 × 10^−5^ cm^2^/s, and 7.14 × 10^−5^ cm^2^/s at 1850 K, 2000 K, 2200 K, and 2500 K, respectively. As shown in Figure 6b, the data are again well fitted ($R^{2}=0.998$) by the Arrhenius equation:

(6) $$D_{Ti}\left(T\right)=3.12\times 10^{-3}\exp\left(-\frac{7.89\times 10^{4}}{RT}\right)$$

### 3.3. Discussion

**Figure 7:**
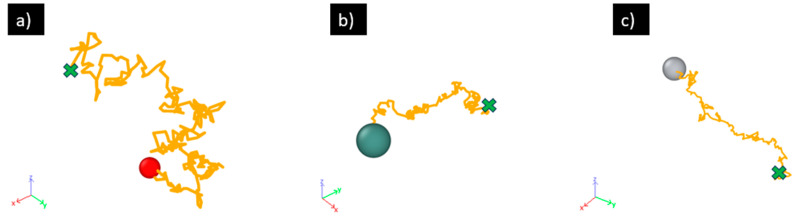
Exemplary diffusion trajectories of O (**a**), Y (**b**), and Ti (**c**) in molten 316L SS at 2500 K from the AIMD simulations. The colored spheres (atoms) mark the starting positions and the green crosses mark the ending positions.

Figure 7 presents exemplary trajectories of the O, Y, Ti in molten 316L SS at 2500 K from the diffusion simulations. They all exhibit the random-walk like Brownian motion characteristic as expected for diffusion in liquids. While diffusion in crystalline solids (e.g., BCC iron) also possesses the random-walk characteristic to a large degree, the diffusion in liquids is more randomized since the atoms are not bound to a crystal lattice and all the atoms, including those in the matrix material (here 316L SS), are undergoing diffusion. 

The data listed in Table 2 and Table 3, as well as the trajectories in Figure 7, suggest that O diffuses faster than Y and Ti in the molten 316L SS. This can be attributed to the smaller size of O (empirical radius: 60 pm) than Y (180 pm) and Ti (140 pm) [40]. Similarly, the Ti-diffusivity is higher than the Y-diffusivity at relative high temperatures such as 2200 and 2500 K, which is also attributable to the smaller size of Ti than Y. However, at lower temperatures such as 1850 and 2000 K, there appears to be a crossover and the Ti-diffusivity falls slightly below the Y-diffusivity. This is the result of the higher activation energy, 7.89 × 10^4^ J/mol of the Ti-diffusion than that of the Y-diffusion, 4.62 × 10^4^ J/mol. It is interesting to point out that the activation energies of O- (1.06 × 10^5^ J/mol), Y- and Ti-diffusion in the molten 316L SS exhibit an inverse correlation with the atomic sizes; the smallest O has the highest activation energy while the biggest Y has the lowest activation energy. This may be understood as resulting from the different level of confinement imposed by the steel matrix on these solute atoms. O, with its small size, is tightly confined by the neighboring steel atoms while Y, with its big size, pushes the neighboring steel atoms out and hence possesses a more open and less confined local environment. However, at temperatures above 2000 K where enough thermal activation is provided for all, the comparison of the diffusivity among the three species is no longer dictated by the activation energy, and instead, it follows the trend of smaller atoms diffusing faster.

As mentioned in the Introduction, due to the experimental and computational challenges in determining the atomic diffusivity at high temperatures, no previous data for O-, Y- or Ti-diffusivity in the molten 316L SS appear to exist in the literature. However, there have been reports by different authors [26,27,28,29,30,31,32] of the calculated diffusivities of O, Y and Ti in BCC iron, which fall in the range of 10^−7^–10^−8^ cm^2^/s, 10^−13^–10^−16^ cm^2^/s and 10^−11^–10^−14^ cm^2^/s, respectively, at temperatures near 1000 K, where the scattering in the data is caused by the different methods used for the calculation. These reported values are all significantly lower than what is obtained in the present study for O-, Y- and Ti-diffusion in molten 316L SS. This is as expected since diffusion in high-temperature liquids is generally much faster than that in a lower temperature crystalline solid (despite the composition difference between 316L SS and pure iron). In the meantime, it is also interesting to notice that the comparison of the diffusivities of O, Y and Ti in BCC iron displays the same ordering as in the molten 316L SS here, i.e., $D_{O}$ > $D_{Ti}\;$> $D_{Y}$ with some overlap/crossover between the latter two. 

The diffusivity of O in molten iron has been previously measured in experiments near the melting point of iron (1538 °C), and the reported data exhibit a fair amount of scattering, ranging from 10^−5^ to 10^−3^ cm^2^/s [22,23,24,25]. For example, Suzuki et al. obtained 2.5 × 10^−5^ to 3.5 × 10^−5^ cm^2^/s in the temperature range of 1560–1660 °C by using a capillary reservoir method and Kawakami et al. acquired ~1.9 × 10^−4^ cm^2^/s at 1550 °C by employing an electrochemical polarization method [22,23]. The diffusivity of O in the molten 316L SS calculated in the present work is 2.89 × 10^−5^ cm^2^/s at 1577 °C (1850 K), which aligns broadly with the literature values for O-diffusivity in molten iron in terms of the order of magnitude [22,23]. 

## 4. Conclusions

In summary, we have used ab initio molecular dynamics simulations to compute the diffusivities of O, Y, and Ti in molten 316L SS, taking advantage of the new on-the-fly machine learning force field feature in the latest VASP software package (version 6.3.2). The diffusivities directly computed at four different temperatures (1850, 2000, 2200 and 2500 K) are further fitted to Arrhenius expressions by which the pre-exponential factors and activation energies are determined for the three species. These expressions can be incorporated in future kinetic models for predicting characteristics (e.g., number density, size distribution) of nanoscale oxide precipitates directly formed during laser-melting-based AM of ODS steels. We have also discussed the relative magnitudes of the diffusivities and diffusion activation energies of O, Y, and Ti in molten 316L SS in terms of their atomic sizes. At temperatures above 2000 K, the magnitude of diffusivity decreases with the increasing atomic size among the three elements, following the order of D_O_ > D_Ti_ > D_Y_. At lower temperatures, cross-over among the three elements occurs on the Arrhenius plot (D vs. 1/T) due to the reverse dependence of the activation energy on the atomic size: a smaller size corresponds to a higher activation energy. Given the lack of experimental data caused by technical challenges in measuring such diffusivities at high temperatures, the results presented here are much needed for the development of the laser-melting-based AM technology for the fabrication of ODS steels. In addition, the ab initio molecular dynamics-based computational methods demonstrated here can be applied to obtain diffusivities in other alloy systems.

## Figures and Tables

**Table 1:** The chemical composition of 316L SS and the numbers of representative atoms in the learning and the application (diffusion) simulations.

	Fe	Cr	Ni	Mn	Si	Mo	C	P	S	Total
Nominal composition (at.%)	Bal	17.90	9.33	1.99	1.46	1.15	0.36	0.08	0.05	100
No. of atoms (for learning)	86	23	12	3	2	1	1	0	0	128
No. of atoms (for diffusion simulations)	292	77	41	9	6	5	2	0	0	432

**Table 2:** The diffusivity of oxygen in molten 316L SS.

T (K)	1850	2000	2200	2500
D_S1_ (10^−5^ cm^2^/s)	2.71	3.93	6.51	12.7
D_S2_ (10^−5^ cm^2^/s)	2.67	5.93	9.85	27.4
D_S3_ (10^−5^ cm^2^/s)	3.28	4.79	6.87	13.5
Average D (10^−5^ cm^2^/s)	2.89	4.88	7.74	17.87

**Table 3:** The diffusivity of Y and Ti in molten 316L SS.

T (K)	1850	2000	2200	2500
Ave. D_Y_ (10^−5^ cm^2^/s)	2.19	2.77	3.39	4.85
Ave. D_Ti_ (10^−5^ cm^2^/s)	1.90	2.63	4.11	7.14

## Data Availability

The data that support the findings of this study are available from the corresponding author upon reasonable request.

## Supplementary Materials

The following supporting information can be downloaded at: https://www.mdpi.com/article/10.3390/ma17071543/s1, Figure S1: Bayesian error estimation (BEE) of energy per atom during machine learning of the force field (with temperature going from 1700 K to 2700 K). The error grows slowly with increasing steps (i.e., temperature) but overall well contained within a few times 10^−5^ eV/atom.
