# Supplementary material for: Atomic Diffusivities of Yttrium, Titanium and Oxygen Calculated by Ab Initio Molecular Dynamics in Molten 316L Oxide-Dispersion-Strengthened Steel Fabricated via Additive Manufacturing
Source: Materials (Basel). 2024 Mar 28;17(7):1543. doi: 10.3390/ma17071543 (PMC11012943; doi:10.3390/ma17071543)
Supplement: Supplementary file 1 [file materials-17-01543-s001.zip › materials-2904428-supplementary.pdf]

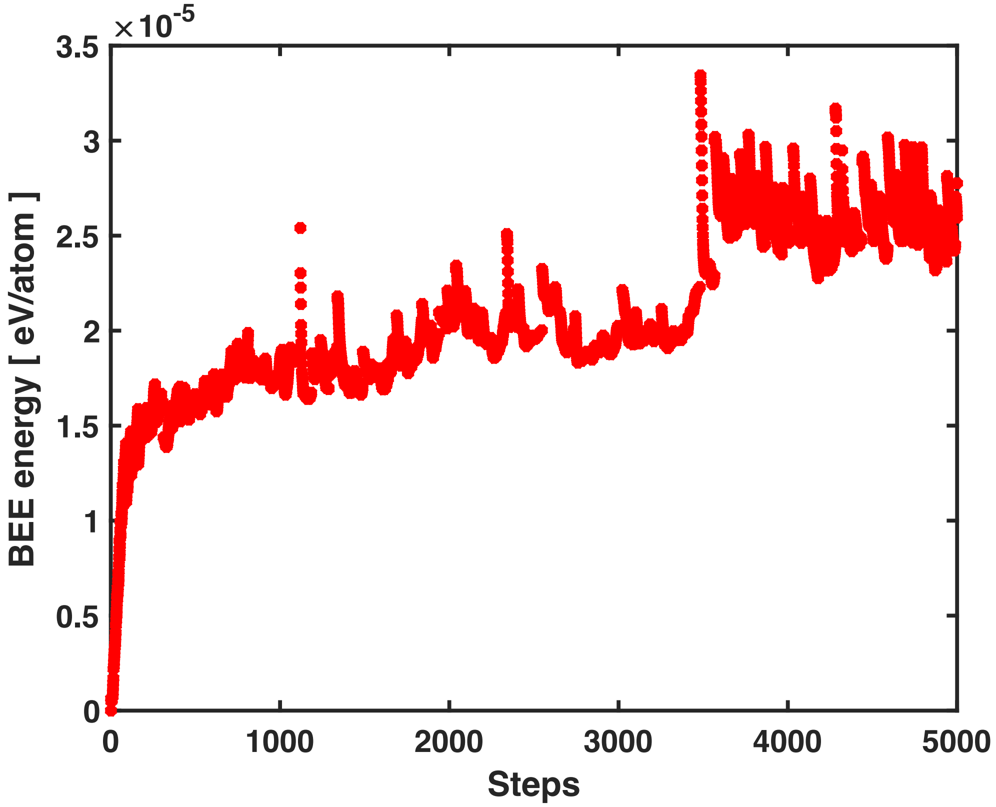

Figure S1. Bayesian error estimation (BEE) of energy per atom during machine learning of the force field (with temperature going from 1700 K to 2700 K). The error grows slowly with increasing steps (i.e., temperature) but overall well contained within a few times 10^{-5} eV/atom.
